# Severe Necrotizing Pneumonia as an Unusual Initial Manifestation of Neurofibromatosis Type 1: A Case Presentation

**DOI:** 10.7759/cureus.106505

**Published:** 2026-04-06

**Authors:** Panagiota Anyfanti, Eleni Marnoutsidou, Afroditi Boutou, Aikaterini Gakidi, Nikolaos Kakaletsis, Theodoros Michailidis, Christina Nousiou, Charalambia Savva, Maria Xanthopoulou, Panagiota Kakotrichi, Eleni Faniadou, Giannis Avgerinos, Konstantinos Dimitrakopoulos, Apostolos Tsapas, Eleni Bekiari

**Affiliations:** 1 Third Department of Internal Medicine, Papageorgiou Hospital, Aristotle University of Thessaloniki, Thessaloniki, GRC; 2 Second Department of Internal Medicine, Hippokration Hospital, Aristotle University of Thessaloniki, Thessaloniki, GRC; 3 Department of Respiratory Medicine, Hippokration Hospital, Thessaloniki, GRC

**Keywords:** lung disease, lung emphysema, necrotizing pneumonia, neurofibromatosis type 1 (nf-1), neurofibromatosis type 1 (nf1), von recklinghausen disease

## Abstract

Neurofibromatosis type 1 (NF1), also known as von Recklinghausen disease, is a relatively common inherited neurogenetic disorder that predisposes individuals to various benign and malignant tumors, primarily in the nervous system. Diagnosis depends on clinical manifestations; however, the clinical presentation of NF1 is highly variable, and complications of NF1 may show great heterogeneity. In this manuscript, we report the case of a 45-year-old male patient who presented with frequent episodes of fever (up to 39°C) and productive cough initially non-responsive to standard-of-care medical treatment. The chest computerized tomography (CT) findings of bullous lung disease, along with skin abnormalities (multiple café-au-lait macules and a plexiform neurofibroma) and iris Lisch nodules, raised the suspicion of undiagnosed NF1. Diagnosis of NF1 was established based on clinical criteria. This case underscores the heterogeneity that may characterize NF1 and demonstrates the diverse and unpredictable nature of the disease. Lung disease may complicate NF1, even as an early yet severe clinical presentation in a previously undiagnosed individual.

## Introduction

Neurofibromatosis type 1 (NF1), otherwise known as von Recklinghausen disease, is a relatively common neurogenetic disorder affecting approximately one in 2500 to one in 3000 people worldwide [1,2]. NF1 is an autosomal-dominant inherited neurogenetic disorder resulting from a germline mutation in the NF1 tumor-suppressor gene on chromosome 17q11.2, which normally produces a protein called "neurofibromin," which regulates cell growth and multiplication [3]. Loss of neurofibromin function predisposes to a series of both benign and malignant tumors, mainly located in the nervous system [4]. In over 90% of cases, café-au-lait macules are present and typically appear within the first two years of life.

Remarkably, the clinical presentation of NF1 is highly variable, and complications of NF1 show a great heterogeneity. Neurofibromas are very common benign tumors in NF1, which typically grow from Schwann cells but usually include endothelial cells, vessels, fibroblasts, or mast cells as well. Cutaneous neurofibromas are the most common form and increase over time without having malignant potential, while plexiform neurofibromas are found in approximately half of the NF1 patients [4-6]. Other common NF1-related malignancies are glioma of the optic pathway, glioblastoma, malignant peripheral nerve sheath tumor, gastrointestinal stromal tumor, breast cancer, leukemia, pheochromocytoma, duodenal carcinoid tumor, and rhabdomyosarcoma [6]. Lisch nodules, well-defined hamartomas of the iris, are specific to NF1 and increase with age without being transformed into malignancy or impacting their vision [4]. Skeletal deformities and cardiovascular abnormalities may also complicate the course of NF1 [6].

Pulmonary involvement in the context of this disease is often neglected and has only recently received attention as part of systemic complications associated with long-term disease. We herein present a case of severe necrotizing pneumonia and notable emphysematic lesions in a young patient as the initial manifestation of a previously undiagnosed NF1.

## Case presentation

A 45-year-old male patient presented to the emergency department complaining of frequent episodes of fever (up to 39°C) and a productive cough for the past three days. His past medical history was unremarkable other than 70-pack-year smoking, and he was not aware of any hereditary diseases in his family. No environmental or occupational exposures of note were reported. Clinically, his vitals were stable (heart rate: 100 bpm, blood pressure: 140/80 mmHg, respiratory rate: 26/min) with an oxygen saturation of 97% (FiO₂ 21%). Physical examination was remarkable for crackles in the right middle zone upon lung auscultation and pigmentary abnormalities of the skin, the largest resembling a nevus-like lesion. White blood cell count and C-reactive protein were substantially elevated (28,000/mm³ and 301 mg/L (reference values <6 mg/L), respectively). A chest X-ray indicated consolidation in the right upper and middle lobes.

**Figure 1:**
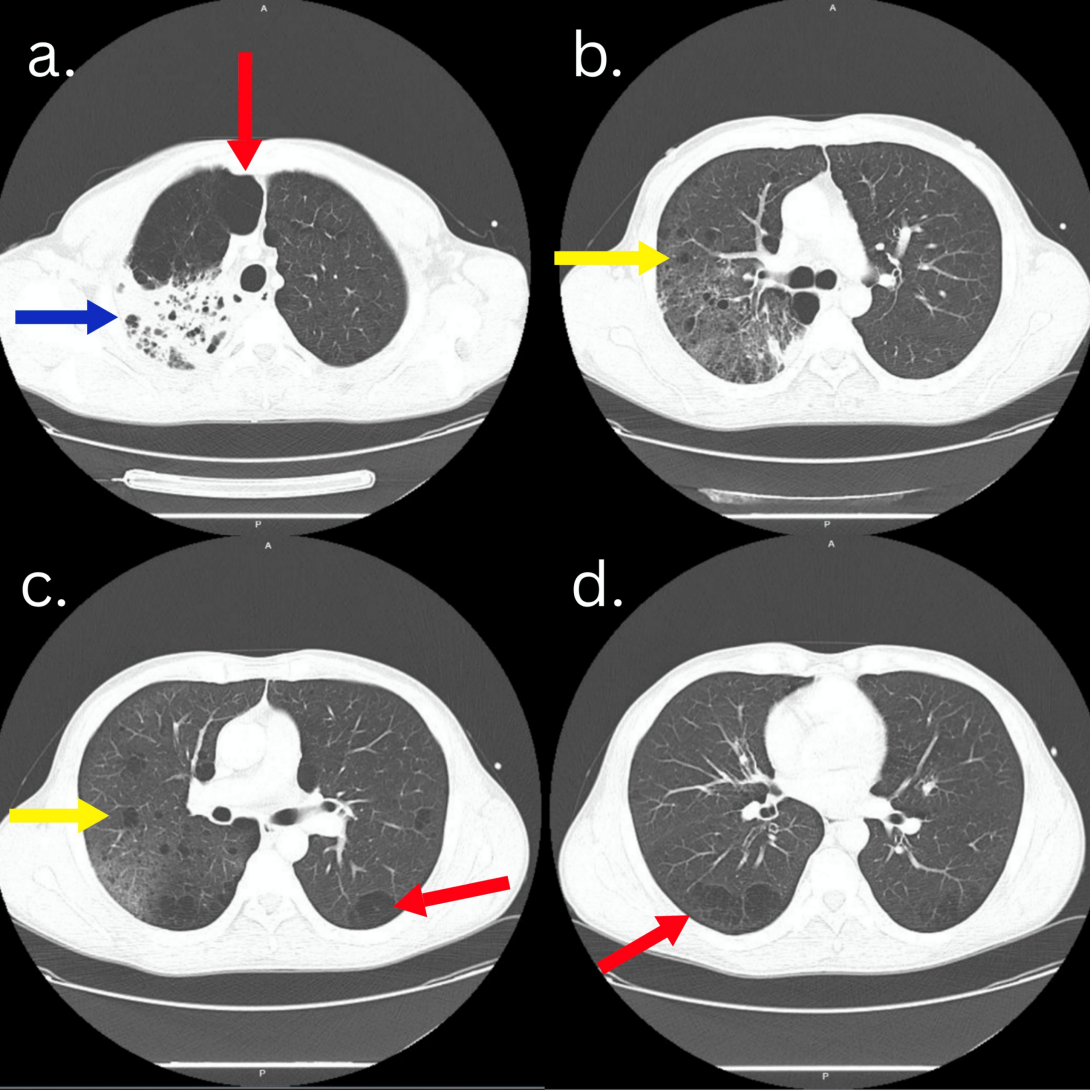
Chest computerized tomography findings. Revealed remarkable centrilobular and subpulmonary pulmonary emphysema (Figure 1a, 1c, 1d; red arrows), multiple pulmonary cystic lesions (Figure 1b, 1c; yellow arrow), and confluent alveolar infiltrates in the posterior part of the right upper lobe consistent with pulmonary microabscesses (Figure 1a; blue arrow).

**Figure 2:**
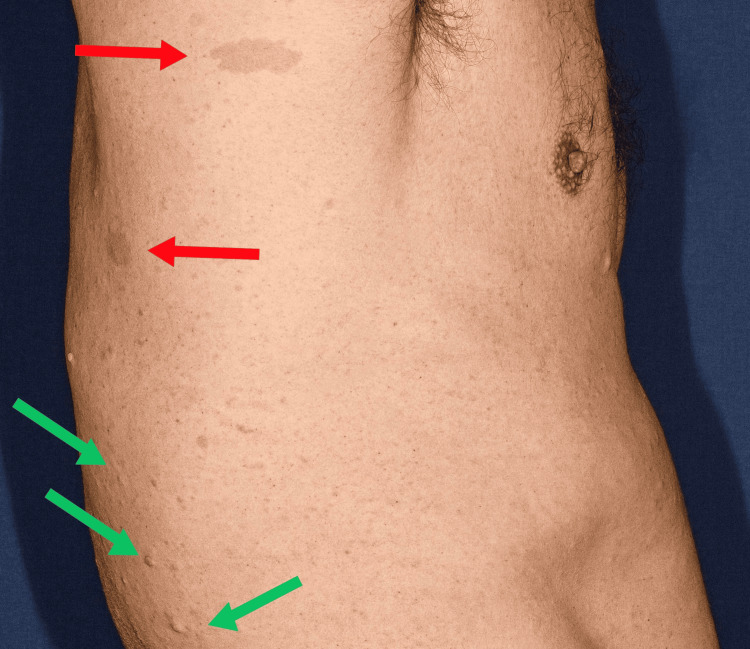
Diffuse pigmentary abnormalities of the skin. Multiple cutaneous neurofibromas, i.e., numerous soft, flesh-colored to light brown papules and small nodules (green arrow), and café-au-lait macules, i.e., well-defined, uniformly light-brown hyperpigmented patches (red arrows), are characteristic cutaneous manifestations of neurofibromatosis type 1 (NF1).

The patient was admitted to the hospital and treated with intravenous ampicillin/sulbactam and azithromycin. Blood cultures for common bacilli, urine antigen tests for *Streptococcus pneumoniae* and *Legionella pneumophila*, and sputum nucleic-acid amplification tests for rapid molecular diagnosis of *Mycobacterium tuberculosis* were all negative. The patient did not respond to treatment, and after 72 hours, antibiotics were escalated to a combination of piperacillin/tazobactam, linezolid, and levofloxacin. Chest computerized tomography (CT) imaging revealed microabscesses in the lungs with a maximum diameter of 12 mm and centrilobular and subpleural emphysematic lesions, the largest bulla corresponding to a maximum diameter of 45 mm, with widespread distribution in the right upper and middle lobes (Figure 1). The CT findings of bullous lung disease in a young individual, along with the pigmentary abnormalities of the skin, raised the suspicion of undiagnosed NF1 (Figure 2). In this diagnostic turn point, an ophthalmologic examination was ordered that revealed the presence of Lisch nodules. Dermatologic examination demonstrated multiple café-au-lait macules and a nevus-like lesion corresponding to plexiform neurofibroma. Diagnosis of NF1 was established based on clinical criteria. The patient gradually responded to treatment with fever resolution following administration of broad-spectrum antibiotics for four days. He was eventually discharged from the hospital after two weeks, following complete resolution of clinical symptoms and normalization of inflammatory markers. The patient and his family were referred to a geneticist for further counseling.

## Discussion

To the best of our knowledge, this is the first case of severe necrotizing pneumonia as the initial manifestation facilitating the diagnosis of NF1 in a young patient. Although NF1 is characterized by a wide range of clinical manifestations, pulmonary involvement has only recently received attention as part of systemic complications associated with the disease. Pulmonary complications can occur in 10-20% of adults with the disease, and these include nodular, bullous, cystic, and/or interstitial parenchymal lesions, with varying degrees of clinical significance and symptomatology [7,8]. However, these have been predominantly described as late complications of NF1 [8,9].

Our patient had been a heavy smoker, but the notable emphysematous lesions in the chest CT raised suspicion of an underlying systemic disease. In particular, the random distribution of cystic changes, the thin but well-defined walls, and the absence of centrilobular arteries support the notion that these pulmonary features are distinct manifestations of NF1 and not secondary to smoking-related emphysema. NF1-associated interstitial lung disease is generally described as bilateral, symmetrical, and predominantly basal. Thin-walled bullae usually coexist and are typically located in the upper lung zones. This combination is not pathognomonic but is regarded as a hallmark pattern of lung involvement in NF1 [10,11]. On the other hand, smoking-related emphysema includes mildly thickened airway walls, and it is typically centrilobular [12].

The diagnosis of NF1 was based on the established diagnostic criteria for individuals without a parent diagnosed with NF1. For those individuals, two or more of the following have to be present: six or more café-au-lait macules over 15 mm in greatest diameter in postpubertal individuals; freckling in the axillary or inguinal region; two or more neurofibromas of any type or one plexiform neurofibroma; optic pathway glioma; two or more iris Lisch nodules identified by slit lamp examination or two or more choroidal abnormalities (CAs) defined as bright, patchy nodules imaged by optical coherence tomography (OCT)/near-infrared reflectance (NIR) imaging; a distinctive osseous lesion such as sphenoid dysplasia, anterolateral bowing of the tibia, or pseudarthrosis of a long bone; and a heterozygous pathogenic NF1 variant with a variant allele fraction of 50% in apparently normal tissue such as white blood cells [13]. Our patient exhibited more than six café-au-lait macules, a plexiform neurofibroma, and iris Lisch nodules, leading to an NF1 diagnosis [13].

The association between NF1 and pulmonary disease is multifaceted, involving both genetic and environmental factors. The variability in pulmonary manifestations among NF1 patients suggests that while smoking can exacerbate lung disease, the underlying genetic predisposition plays a crucial role. NF1 may alter the lung tissue's sensitivity to environmental factors, making NF1 patients more susceptible to severe lung disease upon exposure to smoking. This hypothesis is supported by imaging findings that show minimal interstitial lung disease in never-smokers with NF1, implying that non-smokers might develop lung disease at a later stage and with less severity [14].

## Conclusions

In conclusion, this case illustrates an exceptionally rare presentation of NF1, where severe necrotizing pneumonia was the initial symptom leading to the diagnosis. This case underscores the heterogeneity that may characterize clinical manifestations of NF1, including pulmonary involvement, and demonstrates the diverse and unpredictable nature of the disease as well as the importance of smoking as an exacerbating factor. The purpose of this manuscript is not to generalize findings but to highlight the importance of early recognition of pulmonary manifestations of NF1. Regular monitoring with pulmonary function tests and imaging is warranted for early detection and intervention in NF1-associated pulmonary disease. As this is a single case report, causal relationships cannot be definitively established. However, this case highlights the importance of clinical vigilance to pulmonary manifestations in the context of NF1 that may be severe and may even prompt a patient with unrecognized skin abnormalities to seek medical care, eventually resulting in an NF1 diagnosis.
